# 

**Published:** 1889-12

**Authors:** 


### CONTENTS—DECEMBER, 1889—VOL. 5, NO. 6.

ORIGINAL COMMUNICATIONS—
Maternal Impressions on Fœtus in Utero. By A. N., Denton, M. D . . . . . . . . . . . . . . . . . . . . 201
Malpractice. By Lucius Weinsckenk, Esq., attorney at law, Chicago . . . . . . . . . . . . . . . . . . 208
CORRESPONDENCE—
Acute Gastritis in Children. Letter from Dr. Wilson . 222
Was it Extra-Uterine Pregnancy. Letter from Dr. E. Cross . . . . . . . . . . . . . . . . . . . . . . 223
SOCIETY NOTES—
Address of the Retiring President to the West Texas Medical Society, Dr. P. W. Johns . . . . . . . . 226
Travis County Medical Society Proceedings . . . . . 231
EDITORIAL—
Can an Extra-Uterine Pregnancy Terminate in Natural Delivery? . . . . . . . . . . . . . . . . . . . . . 233
The Texas State Lunatic Asylum . . . . . . . . . . 235
Biography Contemporary Physicians of Texas . . . 236
MEDICAL NEWS AND MISCELLANY . . . . . . . . . . 237
PUBLISHER'S NOTES . . . . . . . . . . . . . . . . . 240

